# Quantitatively Increased Somatic Transposition of Transposable Elements in Drosophila Strains Compromised for RNAi

**DOI:** 10.1371/journal.pone.0072163

**Published:** 2013-08-05

**Authors:** Weiwu Xie, Ryan C. Donohue, James A. Birchler

**Affiliations:** Division of Biological Sciences, University of Missouri, Columbia, Missouri, United States of America; Alexander Flemming Biomedical Sciences Research Center, Greece

## Abstract

In *Drosophila melanogaster*, small RNAs homologous to transposable elements (TEs) are of two types: piRNA (piwi-interacting RNA) with size 23-29nt and siRNA (small interfering RNA) with size 19-22nt. The siRNA pathway is suggested to silence TE activities in somatic tissues based on TE expression profiles, but direct evidence of transposition is lacking. Here we developed an efficient FISH (fluorescence in Situ hybridization) based method for polytene chromosomes from larval salivary glands to reveal new TE insertions. Analysis of the LTR-retrotransposon 297 and the non-LTR retroposon DOC shows that in the argonaut *2* (*Ago2*) and *Dicer 2* (*Dcr2*) mutant strains, new transposition events are much more frequent than in heterozygous strains or wild type strains. The data demonstrate that the siRNA pathway represses TE transposition in somatic cells. Nevertheless, we found that loss of one functional copy of *Ago2* or *Dcr2* increases somatic transpositions of the elements at a lower level depending on the genetic background, suggesting a quantitative role for RNAi core components on mutation frequency.

## Introduction

The genome of *Drosophila melanogaster* contains over 100 families of transposable elements (TEs). The majority of them have potentially functional copies [1]. Studies of the TEs are mostly focused on the genetic consequences of their activities; therefore most attention has centered on germline transposition events. Indeed, the DNA transposon *P* and non-LTR retro-element *I* normally transpose only in the germline [2,3]. However, other TEs are active in somatic tissues. Recently, new transpositions of *hobo* (a DNA transposon) were detected in salivary glands by FISH [4]; and *R2* (non-LTR) has been demonstrated to transpose in various tissues by a PCR-based assay [5].

Interest in somatic transposition is growing after substantial evidence of somatic transpositions of the mammalian non-LTR element L1 (Line 1) was discovered [6]. L1 shows tissue-specific activation: new insertions in neuron precursors (hippocampus) were detected compared to other tissues using an engineered element [7], quantitative PCR [8] or enriched high-throughput sequencing [9]. The expression activation of the retrotransposon is affected by DNA de-methylation [10]. This phenomenon is speculated to be beneficial for brain development [7]. Also, both germline and somatic new L1 insertions were shown to occur during early embryogenesis [11,12]. In that the transposons are regularly restricted by epigenetic mechanisms, the possibility of element activation under conditions that compromise these mechanisms may lead to diseases such as cancer.

In *Drosophila* , TE activities are suppressed in general by small RNA mediated RNA interference [13]. The piRNA pathway is thought to function primarily in the germline and somatic ovarian follicle cells to silence TEs [14]. Thus the main role of the pathway is suggested to maintain genome integrity between generations. On the other hand, the siRNA pathway is assumed to work mainly in the somatic tissues [15]. piRNAs and siRNAs homologous to TEs were isolated via association with the Piwi and Ago2 proteins, respectively [16-19]. When the main components of both pathways are mutated, the expression level of monitored TEs was increased. The *in vivo* mRNA level of selected TEs is elevated several fold when *Dcr2* or *Ago2* is knocked out [17-21]. However, it is not known whether the modest mRNA increase leads to new insertions of the elements. For example, the yeast LTR retrotransposon Ty1 can express to a very high level of mRNA (up to 10% of total mRNA in the host cell), but transposition rate is very low (10^-8^ to 10^-5^ per cell per generation) [22]. The DNA transposon P element is transcribed in somatic cells, but no transposition was observed [2].

A simplified Flourescent In Situ Hybridization (FISH) protocol was developed for polytene chromosomes from salivary glands to demonstrated that new TE insertions do in fact occur as a result of the increased expression when RNAi is compromised. In addition, our results demonstrate that the siRNA pathway represses TE transposition in somatic cells, in addition to the germline, not only in the absence of RNAi but also in a quantitative manner.

## Materials and Methods

### Stocks and genetic crosses

The *Drosophila melanogaster* sequence reference strain *y; cn bw sp* was obtained from the Bloomington Stock center (#2057) and is referred to as 2057. Canton S is a commonly used wild type strain maintained in our lab. The *dcr2* mutant strains *y w eyFLP; FRT42D dcr-2* ^*G173E*^ */CyO* and *y w eyFLP; FRT42D dcr-2* ^*C473Y*^ */CyO* were obtained from R. Carthew’s lab (29). The *ago2* ^414^ strains are *w* ^m4h^ *; ago2* ^*414*^ */TM3, Ser* and *w* ^m4h^ *; ago2* ^*414*^ */MKRS*. Both strains have been maintained in our lab. To cross these mutations into Canton S or 2057 background, we used an multi-balancer strain: *y w* ^67c23^ *; Gla/CyO, P*{*w* ^*+mc*^ *= ActGFP*}*; MKRS/TM3, Ser P*{*w* ^*+mc*^ *= ActGFP*}. The genetic crosses to generate larvae for analysis are listed in Supporting Information (Methods S1).

### FISH protocol

The detailed method can be found in Supporting Information (Methods S1).

### Statistical analysis

We used Fisher exact test and Chi square test for each pair of data sets. We assume that with the same genotype the transposition rate between larvae would be equal and the observed variation was caused by random distribution. For simplicity, we treated each transposition as a single event. The analysis was performed by SAS.

## Results and Discussion

### Development of a method to detect somatic TE transposition events in Drosophila

The polytene chromosomes from *Drosophila* salivary glands display a detailed banding pattern. When a homologous probe for a target sequence is labeled, after *in situ* hybridization, the location of the target sequence can be distinguished within the range of 50-200 kb [23]. Our laboratory developed a very efficient FISH protocol for plant chromosomes [24]. Using that protocol [24], we made changes to published methods for Drosophila chromosomes [23] (see Supporting Information for a detailed protocol).

First, we used a simplified buffer system for squashing and fixing chromosomes. Salivary glands are dissected from 3^rd^ instar larvae in saline solution (0.7% Sodium Chloride), and then transferred to 62.5% acetic acid for fixation and squashing. UV-crosslinking was further applied to adhere the chromatin to the slide. We found that paraformaldehyde used for fixation causes a strong background and is not necessary for FISH. We also improved the technique of squashing to increase the number of cells that are well spread and suitable for analysis.

Second, the probe DNA was prepared by PCR amplification with a size of approximately 1 kb (See Table S1 for primers). Smaller sized DNA fragments (~ 400 bp) for LTRs of the retrotransposons worked well without obvious reduction of signal detection or contributing to the background. These fragments are labeled by incorporating fluorochrome conjugated nucleotide by nick-translation. Using fluorophores replacing biotin labeling permits multiple elements to be analyzed simultaneously.

Third, we used only one buffer (2XSSC+TE) for hybridization. Before hybridization, we denatured the probes and the chromatin on slides in a boiling water bath.

### The distribution of TEs changed in the genome-sequenced reference strain after over two decades of maintenance

We used the genome-sequenced reference strain (Bloomington Drosophila Stock Center is #2057 and referred to as 2057 here) [25] for our experiments. In this strain the location of every element was presumably determined. This known pattern aids in the recognition of the banding pattern and as a test of the efficiency of our FISH protocol.

The LTR-retrotransposon 297 and the non-LTR elements, DOC and F, exhibit increased expression in cells with *ago2* or *dcr2* knockdowns [17-21]. Another non-LTR element, Jockey, has been stable in the sublines of the sequenced isogenic strain including 2057 [26]. Interestingly, for all of these elements, new deletions or insertions occurred during the period of maintenance (Table 1).

**Table 1:** New insertions and deletions detected in 2057.

Element	Total expected	Deletions	Insertions
297	26	9D2 10B1 10D1 28D3 35D3*	74D^#^
DOC	38	32E1 43F1 44C4 49F3 65D1 78E6 84D9 100D1	7C 9C 12B 17B 22B^#^ 27E 57A 66C^#^ 70E^#^ 85D 92A 92A 92D 92F
F	30	34C 41B3 41C6 42A14	32D 33E 47A 48F 85B 89B 98E
Jockey	20		98D^#^

* LTR remained.

# Not present in all larvae and/or only one copy in a genome.

**Figure 1:**
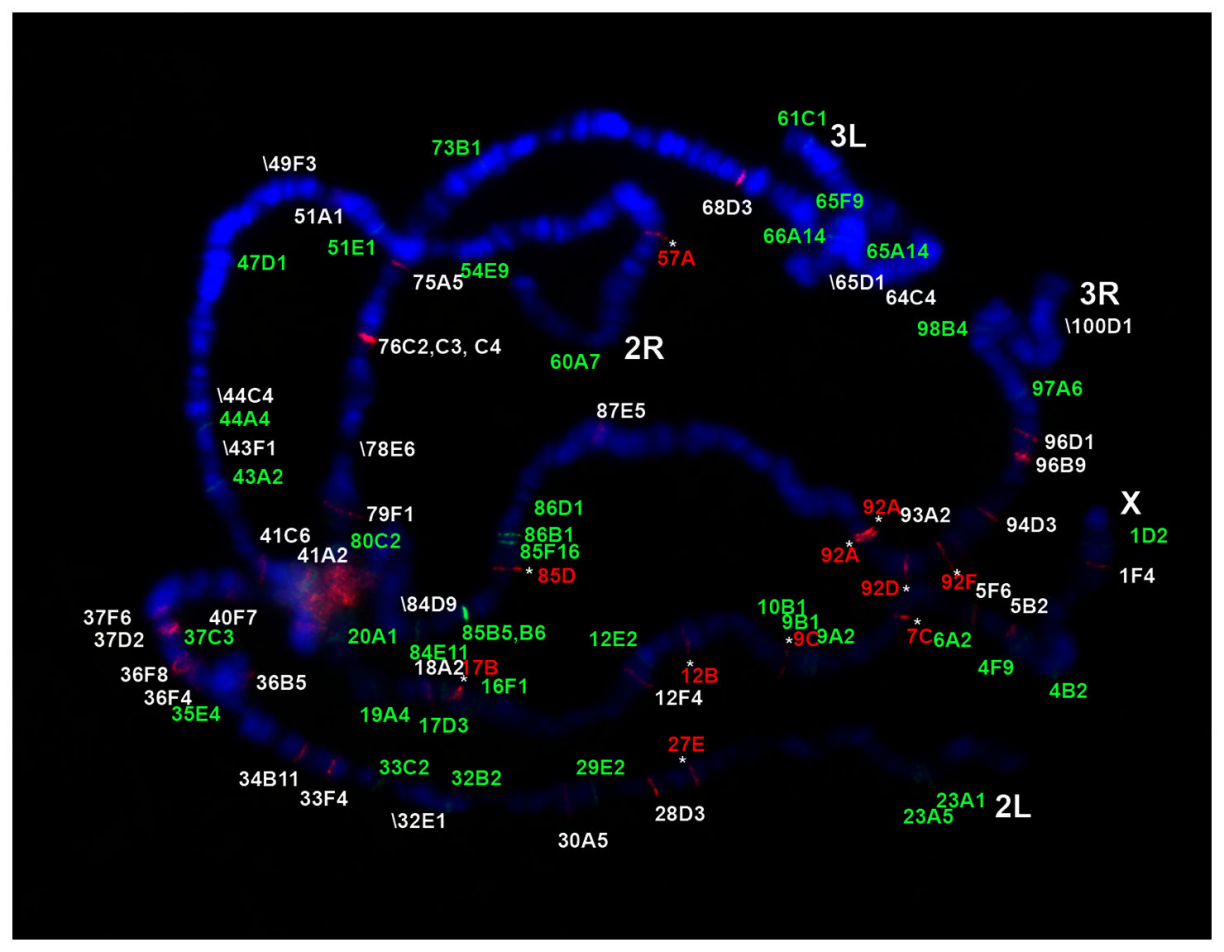
The distribution of 297 and DOC on the chromosomes of 2057. 297 LTR was detected and shown in green and DOC was probed and shown in red. The new bands from the reference genome sequence were indicated with “*”. When an expected band was not present, the name and the position of the band was shown in the picture with the designation: “\” (the deleted 297 bands is shown in Figure S3).

New insertions are expected if the retrotransposons have been activated. Based on the genome sequence, we expected to see 38 bands in the euchromatin for DOC. Eleven new bands were detected (Figure 1). They are likely fixed in 2057 because they are present in all the larvae and their offspring that were analyzed. There are three additional new DOC insertions because they are present in only some of the larvae or their offspring. At the same time, 8 deletions were detected and they are likely fixed in the genome (Figure 1) although it cannot be determined whether such deletion is for all or part of the element that might be below the level of detection. Similarly for the F-element, 7 new bands and 4 deletions were seen, whereas 30 bands were expected (Figure S1). Jockey is more stable in that only one new band was detected with a signal indicating heterozygosity compared to neighboring bands (Figure S2).

For the LTR-element 297, 26 bands were expected. Four bands were deleted and likely fixed (Figure 1). Unlike in plants [27], only a small number of possible solo LTRs were found in the genome, suggesting LTR recombination is not common (Figure S3). No fixed new 297 insertions are detected except one new band detected in one larva, suggesting that 297 is more stable than DOC and F.

Similar results to a few other elements were observed in this strain. Zakharenko et al. [28] analyzed the DNA element hobo and the LTR retrotransposons 412 and *mdg1* using polytene FISH. Out of 30 expected 412 bands, 4 were deleted with 3 novel insertions. For *mdg1*, one deletion and 1 new insertion were found out of 29 expected bands. However, the relatively young element hobo was much more dynamic: 10 of 24 bands were lost from their original locations and 36 new insertions appeared.

### Increased somatic transposition in Dcr2 or Ago2 mutants

**Figure 2:**
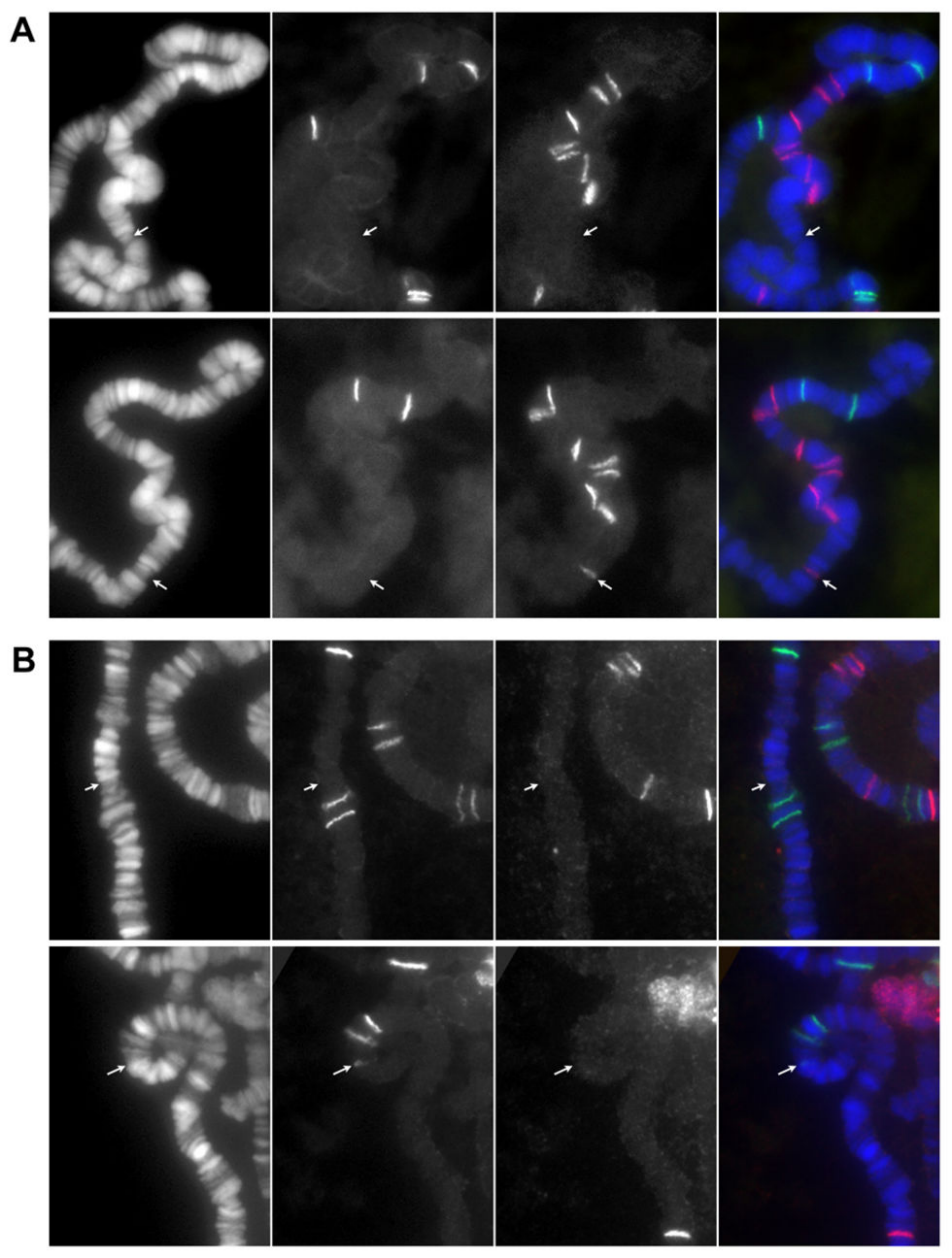
Examples of detected somatic transpositions. (A) A new DOC band is shown at 3R: 88C in a cell of a *dcr2*[CY]/+ larva (bottom row) but not in another cell from the same pair of salivary glands (top row). (B) Similarly a new 297 band is shown at 3R: 86C in a cell of a *dcr2*[GE]*/*[CY] larva but not in its sister cell (upper row). Each row shows three color channels of blue, green and red in order from left to right and a merged image in the very right. The arrows point to the positions of the new bands.

To detect somatic transpositions, salivary glands from only one larva were analyzed on each slide. A TE band present in a cell but not in others on the same slide was defined as a somatic transposition (Figure 2). Also, if a new insertion band is shared by multiple cells from a single larva, this scenario is conservatively scored as one transposition event.

To study how the siRNA pathway affects TE somatic transpositions, we focused on 297 and DOC with both elements being analyzed simultaneously. The *dcr2* (including alleles *dcr2* ^*G173E*^ and *dcr2* ^*C473Y*^ [29], called *dcr2*[GE] and *dcr2[CY]* here) or *ago2* (that is, *ago2* ^414^ [30]) chromosome was introduced into 2057 or the Canton S background by genetic crosses. However, we failed to transfer the *ago2* ^414^ mutation into the 2057 background possibly because the *ago2* ^*414*^ **/+ offspring had very low survival rate. The original *ago2* ^414^ strain was crossed to a strain with balancers *TM3, Ser/MKRS*; the offspring with *ago2* ^*414*^ */TM3, Ser* or *ago2* ^*414*^ */MKRS* were kept as two lines in the lab for several years. The 3^rd^ chromosome from either line with *ago2* ^414^ was designated as *ago2-T* or *ago2-M*, respectively, and both were transferred to the Canton S background to test the effect of *ago2* on TE activities.

**Figure 3:**
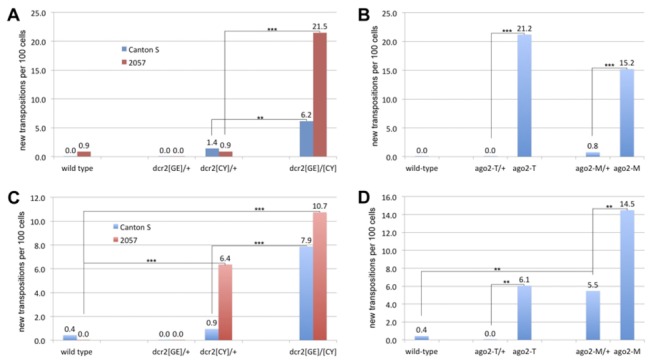
The *ago2* and *dcr2* mutations caused increased transposition of 297 and DOC. The somatic transposition rate of both elements was measured in wild type, heterozygous mutant and homozygous mutant in the 2057 or Canton S backgrounds as the number of events per 100 cells. (A) 297 somatic transposition rate with *dcr2* mutations in both backgrounds. (B) 297 somatic transposition rate with *ago2* mutations in the Canton S background. (C) DOC somatic transposition rate with *dcr2* mutations in both backgrounds. (D) DOC somatic transposition rate with *ago2* mutations in the Canton S background. Paired comparisons for significance of difference are shown in each chart, and “**” indicates p <0.01 and “***” indicates p<0.001.

By analyzing the “+/+” wild type strains 2057 and Canton S, both 297 and DOC were kept largely inert in most of cells: Out of 115 cells in 2057 larvae analyzed, only one 297 somatic transposition and none of DOC were detected (Figure 3A and Table S2). From 231 Canton S cells, none of 297 and one of DOC were found (Figure 3 and Table S3).

In previous studies of RNAi, the *ago2* and *dcr2* mutants were compared to heterozygotes as controls [17-21]. It is assumed that one copy of each wild type gene would be sufficient for full function. 297 remained stable in the *ago2*/+ and *dcr2*/+ strains compared to wild type. It is possible there is a slight increase in the *dcr2[CY]/+* strains, but the difference is not significant in our sample size. Somatic 297 transposition events per 100 cells can reach >20 in the homozygous mutants of *ago2* or *dcr2*, clearly confirming that RNAi regulates TE activities somatically (Figure 3A and 3B and Table S2-4). Most new insertions are detected in only one cell, indicating the transposition occurred late in development. However, occasionally a new event was observed in two or more cells suggesting a transposition during development of the gland. Also, sometimes two new bands are found in a single cell. In a different background, the same mutants can have a significantly different rate of somatic transpositions (*dcr2* in 2057 and Canton S, p <0.0001) (Figure 3A).

DOC is also clearly somatically activated in the *dcr2* or *ago2* strains (Figure 3C and 3D and Table S2-4). Its transposition rate is increased to about 10 per 100 cells. Interestingly, in the heterozygous strain *dcr2[CY]*/+ in the 2057 background, DOC is also significantly activated (6.4 transpositions per 100 cells; p<0.001) whereas for *dcr2[CY]*/+ in the Canton S background, it is only slightly or not active (0.9 transpositions per 100 cells; the difference is not significant compared to the wild type). The strains *ago2-T*/+ and *ago2-M*/+ in the Canton S background are likely identical. Nevertheless, DOC is clearly activated in the *ago2-M*/+ strain (5.5 transpositions per 100 cells) compared to the wild type and in the *ago2*-*T*/+ strain (p<0.001), the rate is comparable to that within the homozygotes of *ago2-T* (6.1 transpositions per 100 cells). Within *ago2-M* homozygotes, DOC transposition is further increased (from 5.5 to 14.5) (Figure 3D). These results indicate that TEs can be activated in heterozygotic RNAi mutant strains, presumably depending on modifying factors.

We noticed in the *dcr2[CY]*/+ strain with the 2057 background, a new insertion of DOC occurred in more than half of the analyzed cells of a larva (25 out of 35). Thus, this transposition likely occurred early in the development of the salivary gland tissue. This finding suggests that somatic transposition could occur in a very early stage.

We also observed possible new insertions in the germline that occurred in *dcr2*/+ flies (Table 2). In the *dcr2*/+ larvae with the 2057 background, new insertions were observed that were shared by all the cells from the same larva but not by other larvae from the 2057 original stock or from other 2057 derived larvae. These cases may represent new germline insertions. Another possibility is that these insertions existed in the original population. However, the population raised in our lab is derived from only several adults. Both elements in wild type are very stable, especially 297; only one new insertion in some individuals was found after more than 2 decades of maintenance. We observed 5 new 297 insertions and 5 new DOC insertions in 4 larvae of *dcr2*[GE]/+. Some of them were shared by individuals in the group but not by the *dcr2[CY]*/+ cohort although siblings from 2057 was used to generate both groups. New germline insertions were also likely for the *dcr2[CY]*/+ group but less frequent. We found 1 new insertion of each element. They were not shared by the wild type control or the *dcr2*[GE]*/+* larvae (Table 2).

**Table 2:** Variant new insertions of 297 and DOC found in wildtype or *dcr2*/+ larvae in the 2057 background.

	larvae #	297	DOC
Wild type			
	l 1		22B 70E
	l 3		70E
	l 6		
	l 3'		
	l 4'	74D	66C
	l 6'		
dcr2[GE]/+			
	l 1	67B 100B	23A 87A
	l 10	100B	23A 62A 87F
	l 15	69D	23A 70E 87A 87F
	l 13'	69D 99F 99A	23A 70E 76A
dcr2[CY]/+			
	l 2		1E
	l 5		1E 66C
	l 6	79F	66C
	l 7		

Analyzing TE transposition events in individual cells has previously been largely performed in single cellular organisms such as yeast *Saccharomyces cerevisiae* or *in vitro* cell cultures. Ty1, the yeast LTR retrotransposon, is possibly the most thoroughly studied element. This TE has over 30 active copies in the yeast genome and is highly expressed with mRNA reaching 10% of total mRNA in the cell [22]. However, the host genome encodes multiple mechanisms to control Ty1’s mobility post-transcriptionally [31]. Consequently, new movements are rarely detected in the cell population, with a maximal rate of <10^-4^ per cell for the engineered element when the most efficient inhibitor gene is knocked out [30]. However, the *Drosophila* LTR element 297 is much more active (as much as 0.2 transposition per cell) in the salivary gland cells when the siRNA pathway is impaired. DOC activity can reach the same high level. The mobility of the L1 element, another non-LTR retro-element, was assayed *in vitro* in the neuroblastoma cell line. New insertions of the engineered element can be detected at a rate of 0.4 per cell [32]. Therefore, it is likely that retrotranspons in somatic cells of multi-cellular organisms have very high potential mobility. This observation highlights the importance of the siRNA pathway and/or other epigenetic mechanisms in maintaining genomic integrity.

## Conclusions

Our results demonstrate, that in the absence of a functional RNAi machinery transposable elements have a significantly increased number of new somatic insertion events. Moreover, when this machinery is compromised by having only half of the normal amount of either Ago2 or Dcr2, there can be an elevated number of somatic insertions. This finding indicates that quantitative modulation of the RNAi machinery under normal circumstances has the potential to impact somatic insertion and hence mutation rate.

## Supporting Information

Figure S1 Distribution of F element on 2057 polytene chromosomes. The unexpected bands from the reference genome sequence were indicated with “*”. When an expected band was not present, the name and the position of the band was still shown with the designation “\”.
(PDF)

Figure S2 Distribution of Jockey on 2057 polytene chromosomes. The unexpected bands from the reference genome sequence were indicated with “*”.
(PDF)

Figure S3 Detecting LTR and full-length bands of 297 on 2057 polytene chromosomes. LTR probe was labeled green and full-length probe is red. The bands detected by the LTR probe but not by the full-length probe are numbered in green font. When an expected band was not present, the name and the position of the band is designated “\”.
(PDF)

Table S1 Somatic transposition summary with *dcr2* mutant and control in 2057 background. (PDF)

Table S2 Somatic transposition summary with *dcr2* mutant and control in Canton S background. (PDF)

Table S3 Somatic transposition summary with *ago2* mutant and control in Canton S background. (PDF)

Table S4 Primers used for amplifying the TE probes. (PDF)

Methods S1 (PDF)

## References

[1] Kaminker JS, Bergman CM, Kronmiller B, Carlson J, Svirskas R et al. (2002) The transposable elements of the *Drosophila melanogaster* euchromatin: a genomics perspective. Genome Biol 3: RESEARCH0084.1-84.2 PubMed: 12537573. 12537573 10.1186/gb-2002-3-12-research0084 PMC151186

[2] Laski FA, Rio DC, Rubin GM (1986) Tissue specificity of Drosophila P element transposition is regulated at the level of mRNA splicing. Cell 44: 7-19. doi:10.1016/0092-8674(86)90480-0. PubMed: 3000622. 3000622 10.1016/0092-8674(86)90480-0

[3] Chaboissier MC, Busseau I, Prosser J, Finnegan DJ, Bucheton A (1990) Identification of a potential RNA intermediate for transposition of the LINE-like element I factor in *Drosophila melanogaster* . EMBO J 9: 3557-3563. PubMed: 1698618. 1698618 10.1002/j.1460-2075.1990.tb07566.x PMC552106

[4] Kovalenko LV, Zakharenko LP, Zakharov IK (2006) Transposition of the hobo element in *Drosophila melanogaster* somatic cells. Russ J Genet 42: 122-128. doi:10.1134/S1022795406020049. 16583701

[5] Eickbush MT, Eickbush TH (2011) Retrotransposition of R2 elements in somatic nuclei during the early development of Drosophila. Mob DNA 2: 11. doi:10.1186/1759-8753-2-11. PubMed: 21958913. 21958913 10.1186/1759-8753-2-11 PMC3190326

[6] Kazazian HH Jr (2011) Mobile DNA transposition in somatic cells. BMC Biol 9: 62. doi:10.1186/1741-7007-9-62. PubMed: 21958341. 21958341 10.1186/1741-7007-9-62 PMC3182954

[7] Muotri AR, Chu VT, Marchetto MC, Deng W, Moran JV et al. (2005) Somatic mosaicism in neuronal precursor cells mediated by L1 retrotransposition. Nature 435: 903-910. doi:10.1038/nature03663. PubMed: 15959507. 15959507 10.1038/nature03663

[8] Coufal NG, Garcia-Perez JL, Peng GE, Yeo GW, Mu Y et al. (2009) L1 retrotransposition in human neural progenitor cells. Nature 460: 1127-1131. doi:10.1038/nature08248. PubMed: 19657334. 19657334 10.1038/nature08248 PMC2909034

[9] Baillie JK, Barnett MW, Upton KR, Gerhardt DJ, Richmond TA et al. (2011) Somatic retrotransposition alters the genetic landscape of the human brain. Nature 479: 534-537. doi:10.1038/nature10531. PubMed: 22037309. 22037309 10.1038/nature10531 PMC3224101

[10] Muotri AR, Marchetto MC, Coufal NG, Oefner R, Yeo G et al. (2010) L1 retrotransposition in neurons is modulated by MeCP2. Nature 468: 443-446. doi:10.1038/nature09544. PubMed: 21085180. 21085180 10.1038/nature09544 PMC3059197

[11] van den Hurk JA, Meij IC, Seleme MC, Kano H, Nikopoulos K et al. (2007) L1 retrotransposition can occur early in human embryonic development. Hum Mol Genet 16: 1587-1592. doi:10.1093/hmg/ddm108. PubMed: 17483097. 17483097 10.1093/hmg/ddm108

[12] Kano H, Godoy I, Courtney C, Vetter MR, Gerton GL et al. (2009) L1 retrotransposition occurs mainly in embryogenesis and creates somatic mosaicism. Genes Dev 23: 1303-1312. doi:10.1101/gad.1803909. PubMed: 19487571. 19487571 10.1101/gad.1803909 PMC2701581

[13] Malone CD, Hannon GJ (2009) Small RNAs as guardians of the genome. Cell 136: 656-668. doi:10.1016/j.cell.2009.01.045. PubMed: 19239887. 19239887 10.1016/j.cell.2009.01.045 PMC2792755

[14] Malone CD, Brennecke J, Dus M, Stark A, McCombie WR et al. (2009) Specialized piRNA pathways act in germline and somatic tissues of the Drosophila ovary. Cell 137: 522-535. doi:10.1016/j.cell.2009.03.040. PubMed: 19395010. 19395010 10.1016/j.cell.2009.03.040 PMC2882632

[15] Golden DE, Gerbasi VR, Sontheimer EJ (2008) An inside job for siRNAs. Mol Cell 31: 309-312. doi:10.1016/j.molcel.2008.07.008. PubMed: 18691963. 18691963 10.1016/j.molcel.2008.07.008 PMC2675693

[16] Brennecke J, Aravin AA, Stark A, Dus M, Kellis M et al. (2007) Discrete small RNA-generating loci as master regulators of transposon activity in Drosophila. Cell 128: 1089-1103. doi:10.1016/j.cell.2007.01.043. PubMed: 17346786. 17346786 10.1016/j.cell.2007.01.043

[17] Czech B, Malone CD, Zhou R, Stark A, Schlingeheyde C et al. (2008) An endogenous small interfering RNA pathway in Drosophila. Nature 453: 798-802. doi:10.1038/nature07007. PubMed: 18463631. 18463631 10.1038/nature07007 PMC2895258

[18] Kawamura Y, Saito K, Kin T, Ono Y, Asai K et al. (2008) Drosophila endogenous small RNAs bind to Argonaute 2 in somatic cells. Nature 453: 793-797. doi:10.1038/nature06938. PubMed: 18463636. 18463636 10.1038/nature06938

[19] Ghildiyal M, Seitz H, Horwich MD, Li C, Du T et al. (2008) Endogenous siRNAs derived from transposons and mRNAs in Drosophila somatic cells. Science 320: 1077-1081. doi:10.1126/science.1157396. PubMed: 18403677. 18403677 10.1126/science.1157396 PMC2953241

[20] Okamura K, Chung WJ, Ruby JG, Guo H, Bartel DP et al. (2008) The Drosophila hairpin RNA pathway generates endogenous short interfering RNAs. Nature 453: 803-806. doi:10.1038/nature07015. PubMed: 18463630. 18463630 10.1038/nature07015 PMC2735555

[21] Chung WJ, Okamura K, Martin R, Lai EC (2008) Endogenous RNA interference provides a somatic defense against Drosophila transposons. Curr Biol 18: 795-802. doi:10.1016/j.cub.2008.07.040. PubMed: 18501606. 18501606 10.1016/j.cub.2008.05.006 PMC2812477

[22] Conte D Jr, Curcio MJ (2000) Fus3 controls Ty1 transpositional dormancy through the invasive growth MAPK pathway. Mol Microbiol 35: 415-427. doi:10.1046/j.1365-2958.2000.01710.x. PubMed: 10652102. 10652102 10.1046/j.1365-2958.2000.01710.x

[23] Biémont C, Monti-Dedieu L, Lemeunier F (2004) Detection of transposable elements in Drosophila salivary gland polytene chromosomes by in situ hybridization. Methods Mol Biol 260: 21-28. PubMed: 15020799. 15020799 10.1385/1-59259-755-6:021

[24] Kato A, Lamb JC, Albert PS, Danilova T, Han F et al. (2011) Chromosome painting for plant biotechnology. Methods Mol Biol 701: 67-96. doi:10.1007/978-1-61737-957-4_4. PubMed: 21181525. 21181525 10.1007/978-1-61737-957-4_4

[25] Brizuela BJ, Elfring L, Ballard J, Tamkun JW, Kennison JA (1994) Genetic analysis of the brahma gene of Drosophila melanogaster and polytene chromosome subdivisions 72AB. Genetics 137: 803-813. PubMed: 7916308. 7916308 10.1093/genetics/137.3.803 PMC1206040

[26] Moschetti R, Dimitri P, Caizzi R, Junakovic N (2010) Genomic instability of I elements of Drosophila melanogaster in absence of dysgenic crosses. PLOS ONE 5: e13142. doi:10.1371/journal.pone.0013142. PubMed: 20957225. 20957225 10.1371/journal.pone.0013142 PMC2949383

[27] Vitte C, Panaud O (2003) Formation of Solo LTRs through unequal homologous recombination counterbalances amplifications of LTR retrotransposons in rice Oryza sativa L. Mol Biol Evol 20: 528-540. doi:10.1093/molbev/msg055. PubMed: 12654934. 12654934 10.1093/molbev/msg055

[28] Zakharenko LP, Kovalenko LV, Mai S (2007) Fluorescence in situ hybridization analysis of hobo, mdg1 and Dm412 transposable elements reveals genomic instability following the *Drosophila melanogaster* genome sequencing. Heredity 9: 525–530. PubMed: 17622267. 10.1038/sj.hdy.6801029 17622267

[29] Lee YS, Nakahara K, Pham JW, Kim K, He Z et al. (2004) Distinct roles for Drosophila Dicer-1 and Dicer-2 in the siRNA/miRNA silencing pathways. Cell 117: 69-81. doi:10.1016/S0092-8674(04)00261-2. PubMed: 15066283. 15066283 10.1016/s0092-8674(04)00261-2

[30] Okamura K, Ishizuka A, Siomi H, Siomi MC (2004) Distinct roles for Argonaute proteins in small RNA-directed RNA cleavage pathways. Genes Dev 18: 1655-1666. doi:10.1101/gad.1210204. PubMed: 15231716. 15231716 10.1101/gad.1210204 PMC478188

[31] Scholes DT, Banerjee M, Bowen B, Curcio MJ (2001) Multiple regulators of Ty1 transposition in *Saccharomyces cerevisiae* have conserved roles in genome maintenance. Genetics 159: 1449–1465. PubMed: 11779788. 11779788 10.1093/genetics/159.4.1449 PMC1461915

[32] Del Re B, Marcantonio P, Capri M, Giorgi G (2010) Evaluation of LINE-1 mobility in neuroblastoma cells by in vitro retrotransposition reporter assay: FACS analysis can detect only the tip of the iceberg of the inserted L1 elements. Exp Cell Res 316: 3358-3367. doi:10.1016/j.yexcr.2010.06.024. PubMed: 20620136. 20620136 10.1016/j.yexcr.2010.06.024

