# Supplementary material for: Quantitatively Increased Somatic Transposition of Transposable Elements in Drosophila Strains Compromised for RNAi
Source: PLoS One. 2013 Aug 5;8(8):e72163. doi: 10.1371/journal.pone.0072163 (PMC3733903; doi:10.1371/journal.pone.0072163)
Supplement: Figure S1 — The unexpected bands from the reference genome sequence were indicated with “*”. When an expected band was not present, the name and the position of the band was still shown with the designation “\”. (PDF) [file pone.0072163.s001.pdf]

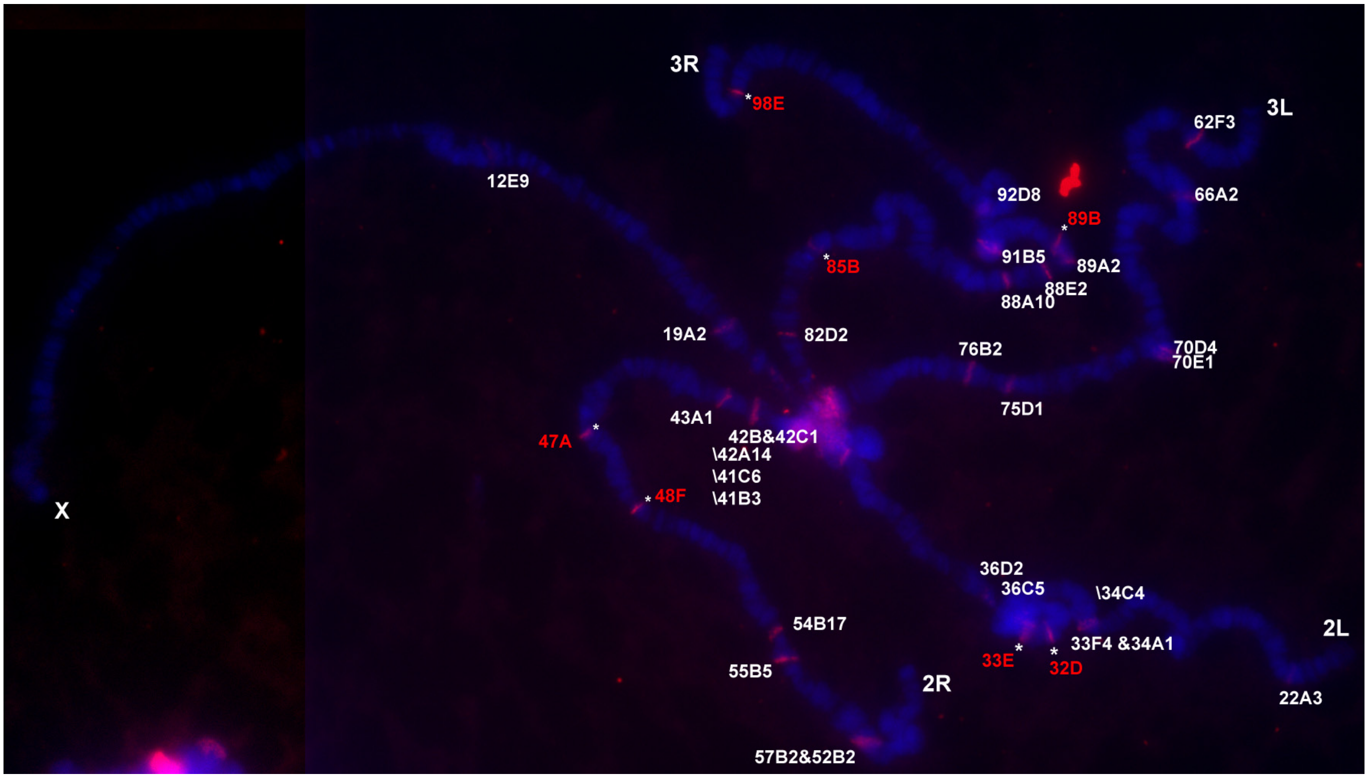

Figure S1. Distribution of F element on 2057 polytene chromosomes. The unexpected bands from the reference genome sequence were indicated with "*". When an expected band was not present, the name and the position of the band was still shown with the designation "\".
