# Supplementary material for: Quantitatively Increased Somatic Transposition of Transposable Elements in Drosophila Strains Compromised for RNAi
Source: PLoS One. 2013 Aug 5;8(8):e72163. doi: 10.1371/journal.pone.0072163 (PMC3733903; doi:10.1371/journal.pone.0072163)
Supplement: Figure S2 — The unexpected bands from the reference genome sequence were indicated with “*”. (PDF) [file pone.0072163.s002.pdf]

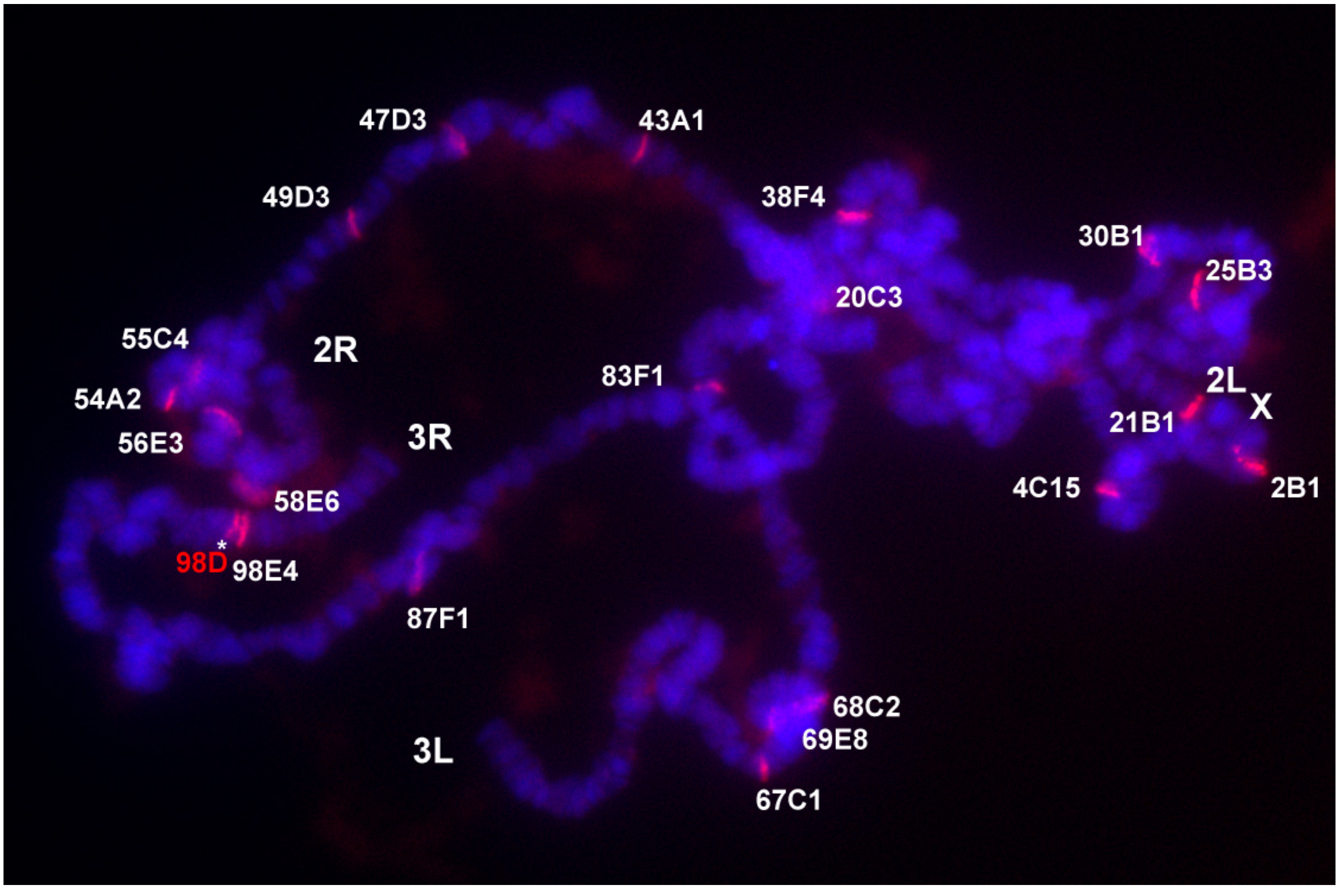

Figure S2. Distribution of Jockey on 2057 polytene chromosomes. The unexpected bands from the reference genome sequence were indicated with "*".
