# Supplementary material for: Quantitatively Increased Somatic Transposition of Transposable Elements in Drosophila Strains Compromised for RNAi
Source: PLoS One. 2013 Aug 5;8(8):e72163. doi: 10.1371/journal.pone.0072163 (PMC3733903; doi:10.1371/journal.pone.0072163)
Supplement: Figure S3 — LTR probe was labeled green and full-length probe is red. The bands detected by the LTR probe but not by the full-length probe are numbered in green font. When an expected band was not present, the name and the position of the band is designated “\”. (PDF) [file pone.0072163.s003.pdf]

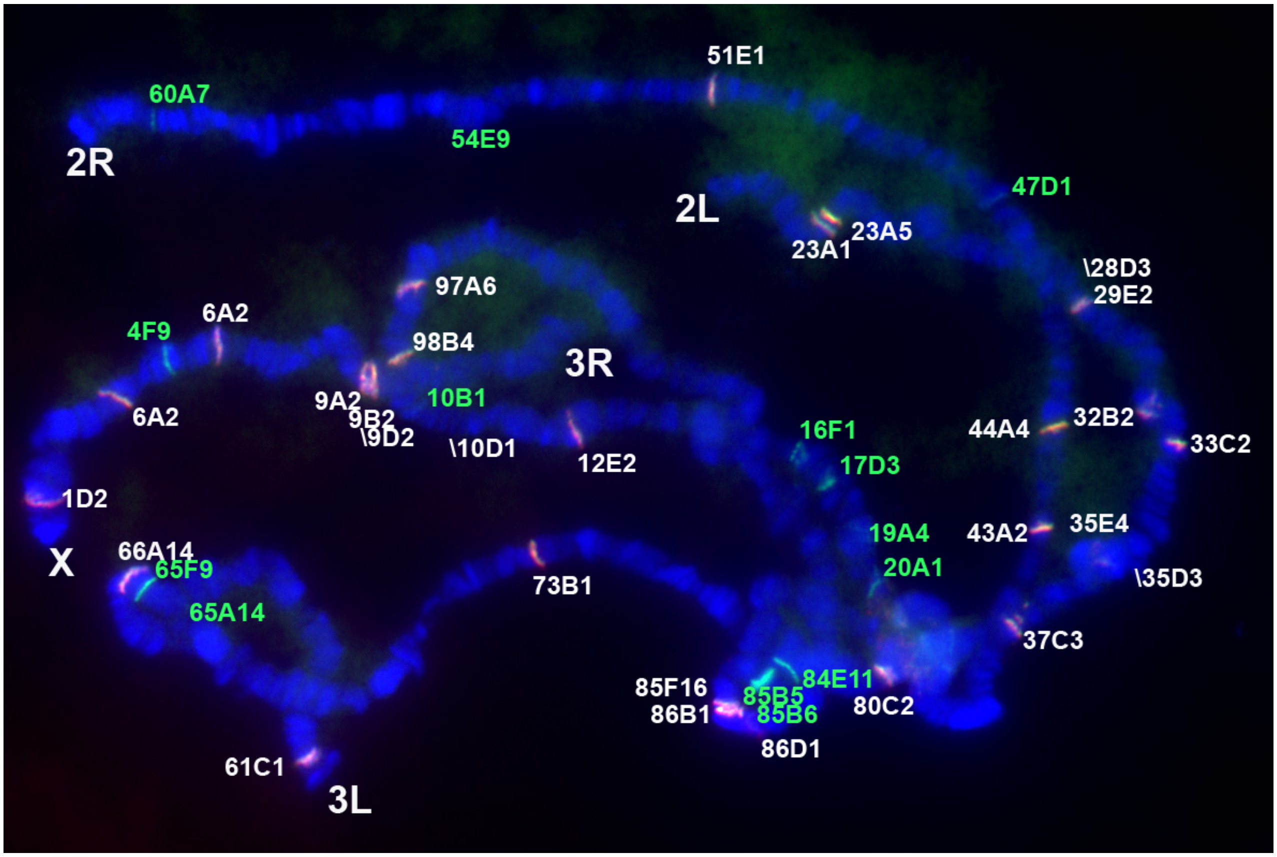

Figure S3. Detecting LTR and full-length bands of 297 on 2057 polytene chromosomes. LTR probe was labeled green and full-length probe is red. The bands detected by the LTR probe but not by the full-length probe are numbered in green font. When an expected band was not present, the name and the position of the band is designated "\".
