# Supplementary material for: Quantitatively Increased Somatic Transposition of Transposable Elements in Drosophila Strains Compromised for RNAi
Source: PLoS One. 2013 Aug 5;8(8):e72163. doi: 10.1371/journal.pone.0072163 (PMC3733903; doi:10.1371/journal.pone.0072163)
Supplement: Table S1 — (PDF) [file pone.0072163.s004.pdf]

Table S1. Somatic transposition summary with *dcr2* mutant and control in 2057 background.

	Larvae	Total cell #	297	DOC
Wild Type				
	l3	35	0	0
	l4	42	1	0
	l6	22	0	0
	l1’	7	0	0
	l3’	2	0	0
	l6’	7	0	0
	Sum	115	1	0
	Rate*		0.9	0.0
dcr2[GE]/+				
	l1	17	0	0
	l10	12	0	0
	l15	21	0	0
	l13’	9	0	0
	Sum	59	0	0
	Rate*		0.0	0.0
dcr2[CY]/+				
	l2	19	0	1
	l5	24	1	2
	l6	35	0	3
	l7	32	0	1
	Sum	110	1	7
	Rate*		0.9	6.4
dcr2[GE]/[CY]				
	l1	48	12	3
	l3	17	4	1
	l6	22	1	4
	l1’	10	2	2
	l2’	24	7	3
	Sum	121	26	13
	Rate*		21.5	10.7

*Number of transpositions per 100 cells
