# Supplementary material for: Quantitatively Increased Somatic Transposition of Transposable Elements in Drosophila Strains Compromised for RNAi
Source: PLoS One. 2013 Aug 5;8(8):e72163. doi: 10.1371/journal.pone.0072163 (PMC3733903; doi:10.1371/journal.pone.0072163)
Supplement: Table S2 — (PDF) [file pone.0072163.s005.pdf]

Table S2. Somatic transposition summary with *dcr2* mutant and control in Canton S background.

	Larvae	Total cell #	297	DOC
Wild Type				
	l1	42	0	0
	l2	54	0	1
	l3	32	0	0
	l5	55	0	0
	l6	48	0	0
	Sum	231	0	1
	Rate*		0.0	0.4
dcr2[GE]/+				
	l2	59	0	0
	l3	26	0	0
	l6	62	0	0
	l5’	30	0	0
	l6’	58	0	0
	Sum	235	0	0
	Rate*		0.0	0.0
dcr2[CY]/+				
	l2	9	0	0
	l3	55	1	0
	l4	24	0	1
	l6	28	0	1
	l1’	34	2	0
	l2’	40	0	0
	l5’	21	0	0
	Sum	211	3	2
	Rate*		1.4	0.9
dcr2[GE]/[CY]				
	l1	23	1	4
	l2	130	7	4
	l1	25	3	6
	Sum	178	11	14
	Rate*		6.2	7.9

*Number of transpositions per 100 cells
