# Supplementary material for: Quantitatively Increased Somatic Transposition of Transposable Elements in Drosophila Strains Compromised for RNAi
Source: PLoS One. 2013 Aug 5;8(8):e72163. doi: 10.1371/journal.pone.0072163 (PMC3733903; doi:10.1371/journal.pone.0072163)
Supplement: Table S3 — (PDF) [file pone.0072163.s006.pdf]

Table S3. Somatic transposition summary with *ago2* mutant and control in Canton S background.

	Larvae	Total cell #	297	DOC
ago2-T/+				
	l1	4	0	0
	l2	7	0	0
	l3	7	0	0
	l4	10	0	0
	l7	11	0	0
	l9	13	0	0
	l11	26	0	0
	l14	16	0	0
	l15	24	0	0
	sum	118	0	0
	Rate*		0.0	0.0
ago2-T				
	l1	21	2	3
	l2	21	5	1
	l3	24	7	0
	sum	66	14	4
	Rate*		21.2	6.1
ago2-M/+				
	l1	16	1	0
	l2	24	0	2
	l4	27	0	3
	l12	10	0	0
	l3	21	0	2
	l5	30	0	0
	Sum	128	1	7
	Rate*		0.8	5.5
ago2-M				
	l1	47	10	6
	l3	20	2	3
	l6	13	3	4
	l11	58	6	7
	sum	138	21	20
	Rate*		15.2	14.5

*Number of transpositions per 100 cells
