# Supplementary material for: Quantitatively Increased Somatic Transposition of Transposable Elements in Drosophila Strains Compromised for RNAi
Source: PLoS One. 2013 Aug 5;8(8):e72163. doi: 10.1371/journal.pone.0072163 (PMC3733903; doi:10.1371/journal.pone.0072163)
Supplement: Table S4 — (PDF) [file pone.0072163.s007.pdf]

Table S4. Primers used for amplifying the TE probes.

297 LTR	GTGACGTATTTGGGTGGTCC
	TGAGTCGAACTAATGTCCCG
297 full-length	GTCGTACCAAAGAAACCGGA
	GGCCCAAACTATGGCAAGTA
DOC	CTTTTCTTTCGTCTCACCGC
	TTGTTTTGAGGGCTGAGCTT
F-element	CAAACTCCAGCTCCTTTTGC
	CGTAACTGCGAAGTCGATCA
jockey	AGAGGTGGCTCTGCAGTCAT
	ACGTCTGTCGGTGTTTTTCC
