# Supplementary material for: Quantitatively Increased Somatic Transposition of Transposable Elements in Drosophila Strains Compromised for RNAi
Source: PLoS One. 2013 Aug 5;8(8):e72163. doi: 10.1371/journal.pone.0072163 (PMC3733903; doi:10.1371/journal.pone.0072163)
Supplement: Methods S1 — (PDF) [file pone.0072163.s008.pdf]

Supporting Information (Methods S1)

Genetic crosses to generate dcr2/+ and dcr2GE/dcr2CY larvae in the 2057 background for FISH. "♀" denotes virgin females. "GFPmw" denotes *P{w^{+mc}=ActGFP}*. "2057" denotes the strain or a corresponding chromosome.

♀ 2057 X ♂ y w^{67c23}; Gla/CyO, GFPmw; MKRS/TM3, Ser GFPmw

↓ ↓

♂ 2057; 2057/Gla; 2057/TM3, Ser GFPmw ♂2057; 2057/CyO, GFPmw; 2057/MKRS

X ♀ 2057 X ♀ 2057

↓ ↓

2057; 2057/Gla; 2057 X 2057; 2057/CyO, GFPmw; 2057

↓

2057; Gla/CyO, GFPmw; 2057

♂ y w^{67c23}; Gla/CyO, GFPmw; MKRS/TM3, Ser GFPmw X ♀ y w eyFLP; FRT42D dcr2/CyO

↓

♂ y w eyFLP; FRT42D dcr2/CyO GFPmw; +/TM3, Ser GFPmw

X

♀ 2057; Gla/CyO, GFPmw; 2057

↓

♂ 2057; FRT42D dcr2/CyO GFPmw; 2057/TM3, Ser GFPmw

X

♀ 2057; Gla/CyO, GFPmw; 2057

↓

2057; FRT42D dcr2/CyO GFPmw; 2057

2057; FRT42D dcr2/CyO GFPmw; 2057 X 2057

↓

Select no GFP late 3rd instar larvae for FISH

2057; FRT42D dcr2GE/CyO GFPmw; 2057 X 2057; FRT42D dcr2CY/CyO GFPmw; 2057

↓

Select no GFP late 3rd instar larvae for FISH

Genetic crosses to generate dcr2/+ and dcr2[GE]/dcr2[CY] larvae in the Canton S background for FISH. "♀" denotes virgin females. "GFPmw" denotes *P{w+mc=ActGFP}*. "CS" denotes the Canton S strain or a corresponding chromosome.

♀ CS X ♂ y w^{67c23}; Gla/CyO, GFPmw; MKRS/TM3, Ser GFPmw

↓ (left) ↓ (right)

♂ CS; Can S/Gla; CS/TM3, Ser GFPmw X ♀ CS → CS; CS/Gla; CS

♂CS; CS/CyO, GFPmw; CS/MKRS X ♀ CS → CS; CS/CyO, GFPmw; CS

CS; CS/Gla; CS X CS; CS/CyO, GFPmw; CS

↓

CS; Gla/CyO, GFPmw; CS

♂ y w^{67c23}; Gla/CyO, GFPmw; MKRS/TM3, Ser GFPmw X ♀ y w eyFLP; FRT42D dcr2/CyO

↓

♂ y w eyFLP; FRT42D dcr2/CyO GFPmw; +/TM3, Ser GFPmw

X

♀CS; Gla/CyO, GFPmw; CS

↓

♂ CS; FRT42D dcr2/CyO GFPmw; CS/TM3, Ser GFPmw

X

♀CS; Gla/CyO, GFPmw; CS

↓

CS; FRT42D dcr2/CyO GFPmw; CS

CS; FRT42D dcr2/CyO GFPmw; CS X CS

↓

Select no GFP late 3rd instar larvae for FISH

CS; FRT42D dcr2[GE]/CyO GFPmw; CS X CS; FRT42D dcr2[CY]/CyO GFPmw; CS

↓

Select no GFP late 3rd instar larvae for FISH

Genetic crosses to generate ago2-T/+ and ago2-T larvae in the Canton S background for FISH. The ago2-M strains were made similarly. "♀" denotes virgin females. "GFPmw" denotes *P{w^{+mc}=ActGFP}*. "CS" denotes the Canton S strain or a corresponding chromosome.

♀ CS X ♂ y w^{67c23}; Gla/CyO, GFPmw; MKRS/TM3, Ser GFPmw

↓ ↓

♂ CS; CS/Gla; Can S/TM3, Ser GFPmw X ♀ CS

↓

CS; CS; CS/TM3, Ser GFPmwX

♂CS; CS/CyO, GFPmw; CS/MKRS X ♀ CS

↓

CS; CS; CS/MKRS

↓

CS; CS; MKRS/TM3, Ser GFPmw

♂ y w^{67c23}; Gla/CyO, GFPmw; MKRS/TM3, Ser GFPmw X ♀ w^{m4h}; ago2^{414}/TM3, Ser

↓

♂ w^{m4h}; Gla/+; ago2^{414}/TM3, Ser GFPmw

X

♀ CS; CS; MKRS/TM3, Ser GFPmw

↓

♂ CS; CS/Gla; ago2^{414}/TM3, Ser GFPmw

X

♀ CS; CS; MKRS/TM3, Ser GFPmw

↓

CS; CS; ago2^{414}/TM3, Ser GFPmw

Self CS; CS; ago2^{414}/TM3, Ser GFPmw

↓

Select no GFP late 3rd instar larvae for FISH

Self CS; CS; ago2^{414}/TM3, Ser GFPmw

↓

Select no GFP late 3rd instar larvae for FISH

Protocol for fluorescent in situ hybridization (FISH) on *Drosophila* salivary gland polytene chromatin

Materials:

Microscope slides (plain, 25x75x1mm, Fisher Scientific)
Cover glass (22x22mm, Fisher)
Cover glass (24x50mm, Corning)
Plastic cover slips (22x22mm, Fisher)
Dissecting dish
200μl pipetter and tips
Dry ice or liquid nitrogen
Metal tray (place a few layers of wet laboratory tissue (Kimwipe) at the bottom to make a humid tray)
Plastic tray (smaller than the metal tray)
Plastic containers with covers (sandwich boxes or similar)

Tools:

Mudont Mini Forceps M5S or Forceps Dumont #5 (Fine Science Tools)
Sharpie Marker pen ultra fine point
Dissecting microscope
Olympus CX21 light microscope
UV cross-linker
Olympus BX61 Fluorescent Microscope equipped with camera Photometrics CoolSNAP™ HQ connected with a personal computer with picture capture software Metamorph

Solutions and Enzymes:

Saline solution: 0.7% NaCl
Fixing solution: 3ml H_2O + 5ml glacial acetic acid
Hybridization buffer: 2x SSC + 1x TE
    20xSSC (1 litre): 175.3g NaCl, 88.2g sodium citrate in H_2O, adjust pH to 7.0 with HCl (14N, a few drops)
    10x TE: 100mM Tris-Cl (pH7.0) + 10mM EDTA pH8.0
For nick translation:
    Non-labeled dNTP mix (2mM)
    DNA polymerase I (10U/μl)
    DNase (100 mU/μl)
    10x Nick translation buffer
    Fluorescein-12-dUTP (green)
    ChromaTide AlexaFluor 594-5-dUTP (red)
Salmon sperm DNA (10mg/ml)
Sodium acetate (3M, pH5.2) or 5M NaCl
95% or 100% ethanol
70% ethanol

1/20 DAPI VectaShield mounting medium (1 vol. of original DAPI medium + 19 vol. of non-DAPI medium)

Procedures:

I. Labeling the probes

DNA fragments used for probes (300bp to 1 kb) can be amplified and column-purified by common methods. Ideally the probe DNA concentration should be greater than 200ng/ul. In case of labeling more diluted DNA probes, one can adjust the total volume of the nick translation reaction and the volume of the 10x buffer accordingly.

1. **Nick translation:** Label 2-5μg of probes as needed. The amount can be adjusted in proportion. For labeling 2 μg probe, add:

DNA	2μg	
Non-labelled dNTPs (2 mM)		2.0ul
Labeled –dNTP (1 mM)		0.5ul
10x Nick translation buffer		2.5ul
DNA polymerase I (10 U/μl)		8.0ul
DNase (100 mU/μl)		0.4ul
H_2O to		25 ul

Mix and incubate at 15°C for 2 hours. After the reaction, the tube can be left at 4°C overnight.

2. **Ethanol precipitation:** Add 20μg autoclaved salmon sperm DNA (10mg/ml) to each 25μl reaction. Add 1/10 volume of 3M sodium acetate or 5M NaCl and 3 volume of 95% or 100% ethanol. Mix. Place the tubes in a -20°C refrigerator for 2 hours to overnight. Spin in a micro-centrifuge at top speed (> 12,000g) for 5-10 min. Remove the supernatant. Wash the DNA pellets with 70% ethanol. Spin for 5 min and remove the ethanol as much as possible. Air dry for 15 min. Dissolve the pellet in 10μl 2x SSC, 1x TE buffer (pH7.0) for each tube. Store at -20°C in dark.

II. Dissecting salivary gland and preparation of slides

1. Pour a thin layer of saline solution in the dissecting dish. Collect late 3rd instar larvae (crawling on the wall of the culture vial) and drop them into the dish.
2. Label the slides. Clean an area of the bench. Put 1-3 cover glasses (22x22mm) on the clean area. Pipet a drop of saline solution (20μl) to the center of each piece of glass.
3. Use two pairs of forceps. Use one to grab the mouth hook (black) and the other one to hold at the position around one third of the body from the bottom. Pull the first pair of forceps and usually the salivary glands and some other parts will be released. Separate the other parts and separate the fat body. Eliminate the fat as much as possible, but a small amount attached to the glands is acceptable.
4. Use the forceps to pick up the pair of glands and drop them in the drop of saline solution on cover glass. Remove the saline solution. Carefully make sure the salivary

glands stay on the glass. Add 20μl of fixing solution. Fix (at room temperature) for 5-8 min. Over-fixation will cause difficulty to squash.

5. Move the cover glass close to the edge of the bench (~10mm away from the edge). Hold the labeled end of the slide upside down. Let the slide touch the edge first and align with the cover glass. Slowly lower the slide to touch the fixing solution and immediately flip the slide to pick up the sample with the cover glass.
6. Place the slide on a clear area of the bench. Cover the slide with two layers of tissue to absorb extra liquid. The cover glass and the sample can be located down the wet area of the covering paper. Use the ultra-fine marker pen (Sharpie) to tap on the cover glass to squash open the cells and spread the chromatin. This type of marker pen has the right weight and even ends (use the bottom end). Start to tap from the edge of the cover slide spiraling into the center and then reverse. Remove the tissue.
7. Check the slide under the microscope to determine if the chromatin is well distributed. Otherwise more tapping is needed.
8. (Optional only if the chromatin is not flattened; be careful this step may make the sample unusable.) Use the end of the marker pen to touch the cover glass where the sample is right underneath when the slide is evenly placed on the bench. Slowly press down the marker pen like applying a stamp with gentle pressure. Check the slide and the chromatin should be well flattened. Repeat.
9. Leave the slide on the surface of dry ice with cover glass and sample facing up for a couple of minutes or longer. The slides can be kept at -80°C for later use.

III. Hybridization

10. Remove the slide from the frozen condition and hold it in air at room temperature for ~30 seconds. The surface of the cover glass will start to thaw. Immediately use a razor blade to lift off the cover glass.
11. Place the slide on a flat cardboard and crosslink using optimal UV program.
12. Turn on the boiling water bath.
13. Prepare the hybridization buffer for each slide:

2xSSC, 1xTE pH7.0	15ul
Labeled probes	0.2ul each

Mix well.

14. Drop 15ul of hybridization buffer right on the top of the sample of each slide. Cover it with a plastic cover slip.
15. Place the slides in a humid tray and then cover the slides with the plastic tray upside down. Put the tray into the boiling water bath and cover it with aluminum foil. Incubate for 5 min to denature the chromatin DNA and the probes.
16. Move the slides to a humid plastic container and seal well. Leave it at 55°C overnight for hybridization.
17. Move the slides to a Coplin jar and pour in pre-warmed 55°C 2xSSC to cover the sample. Incubate the jar at 55°C for 20min. Discard the liquid. Repeat.
18. Remove the slides and shake them gently to remove most of the solution. Place the slides in paper tissue. Add a drop of mounting medium and carefully cover it with a

24x50mm cover glass (avoiding bubbles). The slides are ready for microscopy and can be kept in dark at room temperature for weeks.
